# Biomechanical approaches to understanding the potentially injurious demands of gymnastic-style impact landings

**DOI:** 10.1186/1758-2555-4-4

**Published:** 2012-01-13

**Authors:** Marianne JR Gittoes, Gareth Irwin

**Affiliations:** 1 Cardiff School of Sport, Cardiff Metropolitan University, Cardiff, Cyncoed Road, Cardiff, UK, CF23 6XD

**Keywords:** Impact loading, Laboratory-Based Research, Theoretical Research, Inherent Mechanisms, Regulatory Mechanisms

## Abstract

Gymnasts are exposed to a high incidence of impact landings due to the execution of repeated dismount performances. Biomechanical research can help inform recent discussions surrounding a proposed rule change in potentially injurious gymnastic dismounting. The review examines existing understanding of the mechanisms influencing the impact loads incurred in gymnastic-style landings achieved using biomechanical approaches. Laboratory-based and theoretical modelling research of inherent and regulatory mechanisms is appraised. The integration of the existing insights into injury prevention interventions studies is further considered in the appraisals. While laboratory-based studies have traditionally been favoured, the difficulty in controlling and isolating mechanisms of interest has partially restricted the understanding gained. An increase in the use of theoretical approaches has been evident over the past two decades, which has successfully enhanced insight into less readily modified mechanisms. For example, the important contribution of mass compositions and 'tuned' mass coupling responses to impact loading has been evidenced. While theoretical studies have advanced knowledge in impact landing mechanics, restrictions in the availability of laboratory-based input data have suppressed the benefits gained. The advantages of integrating laboratory-based and theoretical approaches in furthering scientific understanding of loading mechanisms have been recognised in the literature. Since a multi-mechanism contribution to impact loading has been evident, a deviation away from studies examining isolated mechanisms may be supported for the future. A further scientific understanding of the use of regulatory mechanisms in alleviating a performer's inherent injury predisposition may subsequently be gained and used to inform potential rule changes in gymnastics. While the use of controlled studies for providing scientific evidence for the effectiveness of gymnastics injury counter measures has been advocated over the past decade, a lack of information based on randomised controlled studies or actual evaluation of counter measures in the field setting has been highlighted. The subsequent integration of insight into biomechanical risk factors of landing with clinical practice interventions has been recently advocated.

## Review

### Introduction

Gymnastic-style landings involving high-velocity impacts and controlled rotation during ground contact are performed regularly in sport e.g. during landing from a vertical jump or in dismounting from a gymnastics apparatus. Gymnasts are naturally exposed to a high frequency of impact landings and may be required to perform dismounts in excess of 200 times a week [1]. Unlike many other sports involving impact landings, gymnastic routines uniquely require a simultaneous address of performance and injury objectives. In dismounting, gymnasts are challenged by the need to modulate a prescribed rotation of the body orientation in flight to ensure the feet contact the ground. For example, when dismounting from the beam apparatus, gymnasts are frequently required to prepare for landing following a backward or forward somersault (rotation about the transverse axis) performed with high degrees of hip flexion (piked position). The subsequent ground contact or impact landing phase must be achieved using a safe, aesthetic and well-executed, double-foot landing. Although performed less frequently, single-foot impact landings such as performed in a floor routine, require similar performance and injury objectives to be addressed but typically require a succeeding skill to be performed.

Constraints in the ability of a gymnast to satisfy the multiple requirements of competitive landing tasks have subsequently been linked to errors in performance and high injury incidence rates [2]. Performance deductions may, for example be incurred for the execution of an uneven landing involving the use of multiple, single-foot placements during the impact landing phase. A serious problem faced by modern-day gymnasts is however the subsequent injury risks associated with competitive landing tasks. In 1983, Hunter and Torgan [3] questioned the need to re-evaluate gymnastic scoring of dismounts following the high incidence of associated major acute knee injuries e.g. tears to the anterior cruciate ligament. Caine and colleagues [4] highlighted that 36% of all injuries sustained by young competitive females occurred during dismounting. Singh and colleagues [5] more recently confirmed that gymnastics had one of the highest injury rates for all girls' sports between 1999 and 2005 and reported a high proportion of acute strain/sprain (44.5%) and fracture/dislocation (30.4%) diagnoses within the respective cohort. While contemporary epidemiological studies of gymnastic-related injuries have remained sparse, discussions for rule changes to de-emphasise 'sticking' landing routines in the scoring of dismounts have remained evident in the biomechanics literatures [e.g. [6]].

The ability of a performer to resist the collapse of the lower extremities has been suggested to influence the success with which reaction forces are attenuated [2]. Performers are exposed to rapidly occurring and high magnitudes of ground reactions forces during impact landings typically performed in gymnastic. Peak vertical ground reaction forces exceeding nine bodyweights and occurring in less than 0.05 s have been reported by McNitt-Gray and colleagues [2] for drop landings (height: 1.82 m) performed by gymnasts. Biomechanical analyses of the loading mechanisms used in double- and single-foot impact landings provide scientific support for the physical demands incurred on the gymnastic performer. Quantification of the physical demands imposed in landing may subsequently help to inform the respective discussions surrounding a potential rule change in gymnastic dismounting. The aim of this review was to appraise the development of current understanding of the loading mechanisms influencing the potentially injurious demands of gymnastic-style impact landings using biomechanical approaches. The review typically appraised existing insights into double-foot impact landings in order to assist the discussions regarding regulatory changes in commonly performed gymnastics dismounts.

### Loading mechanisms

As evidenced in Table 1, biomechanical investigations of commonly-performed gymnastic-style impact landings have endeavoured to enhance insight into the inherent and regulatory mechanisms that can influence loading and the physical demands incurred. Multiple innate mechanisms including a performer's lower leg alignment [7], neuromuscular control [8], knee joint musculature [9-11] and joint laxity [12] have been linked to injury predisposition and have further been accounted for by gender differences. Gender disparities in the incidence of impact landing injuries have additionally been attributed to athletic posture and movement patterns [13], which may be modified or regulated by the performer. As a partial consequence of the voluntary or sub-conscious adaptations that can be achieved and integrated into safe and effective landing protocols, insights into regulatory loading mechanisms have typically been widespread in the literature. Studies of regulatory mechanisms have examined landing experience [14], impact velocity or height [2,14,15], technique [16-18] and the nature of the impacting interface [2]. While some mechanisms have been consistently recognised as contributors to injury predisposition in impact landings, less well documented mechanisms such as a performer's inherent mass composition [19,20] and lower extremity stiffness response, which was defined as the relationship between the deformation of a body and a given force [21], have recently emerged in the literature. The most prominent mechanisms contributing to impact loading have however been difficult to ascertain due to the frequent examination of isolated mechanisms and the use of diverse research approaches and analyses.

**Table 1:** Summary of biomechanical studies of loading mechanisms in impact landings

Mechanism	Study	Study type	Load estimation approach	Landing task protocol	Landing height		Key loading response finding(s)
**Technique**							

*Knee joint flexion*							

	[15]	Laboratory	Inverse dynamics	Drop (Double-foot)	0.15-1.05 m		Inverse relationship between initial knee flexion & peak GRFr

	[16]	Laboratory	Inverse dynamics	Drop (Double-foot)	0.59 m		Inverse relationship between maximum knee flexion & GRFv

	[23]	Laboratory		Drop (Double-foot)	0.80 m & 1.15 m		Higher (32%) GRFv with stiff than soft knee (0.80 m, hard mat)

	[25]	Laboratory	Inverse dynamics	Drop (Double-foot)	0.32 m, 0.62 m,1.03 m		Direct relationship between knee stiffness & peak GRFv

	[26]	Laboratory	Spring-mass assumption	Drop (Double-foot)	0.30 m (12 inch)		Higher GRFv (55%) with stiff than soft (bent) knee

	[27]	Laboratory	Simulation modelling		0.10 m-0.40 m		Non-linear, inverse relationship between knee flexion & peak GRFv

	[51]	Theoretical		Drop (Double-foot)	0.46 m		Change in peak GRFv (1.5 BW) with modified knee flexion timing

*Foot placement*							

	[17]	Laboratory	Inverse dynamics & electromyography	Drop (Double-foot)	0.40 m		Higher (3.4 times) GRFv impact peak in HTL than FFL

	[18]	Laboratory		Drop (Double-foot)	0.30 m		Unreported kinetic measures

*Gender-specific*							

	[11]	Laboratory	Electromyography	Drop (Double-foot)	0.52 m		No gender difference in peak GRFv or IGRF at 50 & 100 ms

	[18]	Laboratory		Drop (Double-foot)	0.30 m		Unreported kinetic measures

	[28]	Laboratory	Inverse dynamics	Drop (Double-foot)	0.60 m		No gender difference in magnitude, time and rate of peak GRFv

	[30]	Laboratory	Inverse dynamics	Stop-jump	Not reported		Higher peak GRFv (24%) in females than males

	[31]	Laboratory	Inverse dynamics	Drop (Single-foot)	0.30 m		Higher peak GRFv (9%) in females than males

	[34]	Laboratory	Inverse dynamics	Stop-jump	Not reported		Higher knee extension & valgus moments in females than males

	[35]	Laboratory	Inverse dynamics	Drop (Double-foot)	0.60 m		Higher peak GRFv (34%) in females than males

**Landing height**							

	[2]	Laboratory		Drop (Double-foot)	0.69 m, 1.25 m, 1.82 m		Positive relationship between landing height & peak GRFv

	[14]	Laboratory		Drop (Double-foot)	0.32 m, 0.72 m, 1.28 m		Positive relationship between landing height & peak GRFv

	[15]	Laboratory	Inverse dynamics	Drop (Double-foot)	0.15-1.05 m		Exponential relationship between landing height & peak GRFr

	[23]	Laboratory		Drop (Double-foot)	0.80 m & 1.15 m		Unreported kinetic measures

	[24]	Laboratory		Drop (Double-foot)	0.30 m, 0.60 m, 0.90 m		No reported statistical comparison between heights

	[25]	Laboratory	Inverse dynamics	Drop (Double-foot)	0.32 m, 0.62 m,1.03 m		Positive relationship between landing height & peak GRFv

	[27]	Laboratory	Spring-mass assumption	Jump (Double-foot)	0.10 m-0.40 m		Exponential relationship between landing height & peak GRFv

**Impacting interface**							

	[2]	Laboratory		Drop (Double-foot)	0.69 m, 1.25 m, 1.82 m		No difference in peak GRFv between mat stiffness

	[23]	Laboratory		Drop (Double-foot)	0.80 m & 1.15 m		No difference in peak GRFv between mat stiffness

	[46]	Theoretical	Simulation modelling	Drop (Double-foot)	0.43 m		Peak GRFv sensitivity to heel pad stiffness

**Performer experience**							

	[14]	Laboratory		Drop (Double-foot)	0.32 m, 0.72 m, 1.28 m		

	[24]	Laboratory		Drop (Double-foot)	0.30 m, 0.60 m, 0.90 m		Higher GRFv in gymnasts than recreational athletes (0.60 & 0.90 m)

**Landing task**							

	[32]	Laboratory		Backward rotating tuck & pike (beam)	2.18 m & 2.22 m		Unreported kinetic measures

		Laboratory	Inverse dynamics & electromyography	Drop, front & back tucked salto (beam)	0.72 m		Between task differences in net joint moments after contact

	[48]	Theoretical	Simulation modelling	Backward & forward rotating somersault (vault)	Not reported		Reduced peak GRFv & GRFh in tasks using optimised strategies

**Mass composition**							

	[19]	Theoretical	Simulation modelling	Drop (Double-foot)	0.43 m		Peak GRFv (24.3 bodyweights) attenuated by soft tissues

	[20]	Theoretical	Simulation modelling	Drop (Double-foot)	0.46 m		Peak GRFv sensitivity to soft & rigid mass composition

	[46]	Theoretical	Simulation modelling	Drop (Double-foot)	0.43 m		Higher peak GRFv (13%) with higher bone mass (20%)

	[49]	Theoretical	Simulation modelling	Drop (Double-foot)	0.46 m		Peak GRFv (8.6 bodyweights) attenuated by soft tissues

**Mass coupling**							

	[20]	Theoretical	Simulation modelling	Drop (Double-foot)	0.46 m		Subject-specific GRFv response to coupling parameter changes

	[47]	Theoretical	Simulation modelling	Drop (Double-foot)	0.43 m		Insensitivity in peak GRFv to coupling parameters

GRFr: Peak resultant ground reaction force, GRFv: Peak vertical ground reaction force, GRFvh Peak horizontal ground reaction force IGRF: ground reaction force impulse, HTL: heel-toe landing, FFL: forefoot landing

### Laboratory-based studies of loading mechanisms

The majority of biomechanical investigations of impact landings have utilised laboratory-based approaches that have provided descriptive insights into regulatory loading mechanisms. While the laboratory-based approaches have included, practical hypothesis-driven experimental studies, the majority of studies have been observational in nature. As highlighted by Yeow and colleagues [15], many previous studies had specifically used motion analyses to examine various landing conditions e.g. diverse heights [14,15,22-24], lower extremity landing technique [15,16,25-27], experience [14,22,24] and the nature of the impacting interface [2,23]. Laboratory-based studies examining regulatory changes to landing technique have been the most evident in the literature. Decker and colleagues [28] reported that it has generally been accepted that the internal and external loads experienced in landing may be manipulated by the lower extremity kinematics (technique). While investigations of self-selected techniques have established a common phasic joint-by-joint reduction in whole body momentum [14,16,19,29], studies identifying individual and marginal ankle, knee and hip joint kinematic adjustments have suggested contradictory effects on the resulting impact loads [2,15,30,31]. Other studies [15,16,23,25-27] examined the effects of diverse degrees of knee joint flexion during ground contact using controlled comparisons of 'stiff' and 'soft' landing techniques, which were typically differentiated by the maximum knee flexion permitted on initial ground contact. As illustrated in Table 1, an inverse relationship between the degree of initial or maximum knee flexion and the resulting peak ground reaction force experienced has been commonly reported in the respective studies. As a consequence of the need to execute prescribed landing techniques to achieve high dismount scores in gymnastics, biomechanical studies implementing a restricted rather than self-selected knee joint motion may better examine the demands of a gymnastic performer.

Further research has examined the associative effects of multiple regulatory loading mechanisms. Studies investigating the interaction of landing height with experience [14,22,24] or the nature of the impacting interface [22,23] have been prominent but a continuing lack of laboratory-based data regarding high landing heights has been reported [15]. As evidenced in Table 1, laboratory-studies involving controlled drop landings have been common and have typically examined landing performed from heights of less than 1.50m without prior flight phase rotation. Studies examining 'real' gymnastic-style landings e.g. Gittoes and colleagues [32], have however highlighted that realistic landing heights for gymnastic dismounts typically exceed 2.00m. While some insight into more complex gymnastic-style landings involving flight phase rotation has been achieved in the literature [32,33] limited understanding of the regulation of loading in more challenging dismounts continues to exist. Since, gymnastic dismounts are typically characterised by: 1. a requirement to gain height in flight; 2. a need to control whole body orientation in landing and 3. an exacerbated lower limb injury risk, extended insight into challenging 'realistic' height conditions and more complex gymnastic landing manoeuvres is warranted.

Within gymnastics routines, females typically have a shorter time in the air and subsequently gain less height than males. In order to achieve higher scores, females continue to attempt similar transverse (somersaulting) and longitudinal (twisting) rotations to their male counterparts, which potentially accentuates the physical demands experienced by females. The heightened injury predisposition of females performing impact landings has primarily been addressed by an extensive body of research examining gender-related mechanical responses [18,28,31,34,35]. Contradictory findings regarding gender-based techniques have however limited understanding of the primary loading mechanisms underpinning impact landings. Gender comparisons of the techniques employed in double-foot drop jump landings have suggested a tendency for the use of a greater range of motion in the knee (20%) [28] and ankle (up to 39%) [28,35] by females compared to males. Cortes and colleagues [18] contrastingly suggested a lack of gender differences in lower extremity joint flexion angles during the ground contact phase of double-foot drop landings performed utilising diverse foot placement strategies.

In contrast to studies examining landing technique, investigations of gender-based loading responses in single- [31] and double-foot [35] have frequently confirmed heightened lower extremity loading in females compared to males. Females have been suggested to be predisposed to larger peak vertical ground reaction forces of between 9% [31] and 34% [35] when compared to their male counterparts. The current lack of consensus regarding the gender-based landing technique and loading responses predisposing females to injury in impact landings inhibits the extent to which gender-based prevention strategies may be developed and implemented. The preference for kinematic analyses alone and diversity in the protocols investigated in gender-based studies may partially contribute to the incomplete insight gained in the literature. Further insight into the gender-disparity may subsequently be achieved by the wider application of more comprehensive biomechanical analyses. As evidenced by Decker and colleagues [28], examinations of internal joint kinetics have been important in quantifying loads for establishing gender-based control strategies during drop landings. Future studies that further examine the interaction of gender-based technique modifications and the resulting internal and external loads may therefore be warranted in the literature.

Quantification of the joint moments of force produced at the ankle, knee and hip have provided a valuable understanding of the internal loads, joint-specific stresses and controlling mechanisms used to decelerate the body in landing [36]. Inverse dynamics, which integrates kinematic, external force and inertia data collected in a laboratory based setting with a linked-segment assumption, has been the preferred tool for the estimation of internal joint loading. While employing inverse dynamic analyses, DeVita and Skelly [16] established a 19% greater absorption of the body's kinetic energy in a soft landing (less than 90° knee flexion) compared to a stiff landing (greater than 90° of knee flexion). While also using inverse dynamic analyses, Kovacs and colleagues [17] reported greater relative knee joint contributions to the total lower extremity torque produced using a heel-toe (45%) compared to a forefoot (37%) landing technique. However, Kovacs and colleagues [17] further recognised the need to examine individual muscle contributions, which are precluded in inverse dynamic analyses, through the use of electromyography. More recent laboratory-based studies continue to advocate the use of inverse dynamics [e.g. [15]] and electromyography [e.g. [11]] for the estimation of internal loading in dynamic movements. Although potentially limited by their descriptive nature, laboratory-based studies continue to remain popular in biomechanical investigations of landing and are becoming more evident in a growing body of research examining the influence of applied injury prevention strategies.

### Application of laboratory-based studies to injury prevention strategies

As suggested by Daly and colleagues [6] in 2001, controlled trials may provide the best scientific evidence for the effectiveness of gymnastics injury counter measures, but a lack of information based on randomised controlled studies or actual evaluations of counter measures in the field setting existed. While the use of a 'correct technique' had been considered essential to prevent gymnastics injuries in landing [6], an explicit link between adapted landing techniques and an alleviation of the high incidence of gymnastic-style landing injuries had been difficult to ascertain in the literature. As further evidenced by the contradictory outcomes of gender-based studies, establishing common responses to loading mechanisms, that may inform injury prevention programs, has proven difficult. In 2000, Boden and colleagues [13] had however suggested that improved jumping, stopping and turning techniques had shown promising results in injury prevention programs.

A number of recent studies have attempted to identify biomechanical predictors of landing injuries for prevention interventions [37-39]. 'Clinician friendly' approaches for predicting anterior cruciate ligament injuries in impacts using biomechanical measures such as knee flexion range of motion have been identified [37,39]. However, relatively less attention has been given to establishing the short- and long-term effectiveness of injury prevention interventions. As suggested by Daly and colleagues [6] over a decade ago, continuing biomechanical research into the mechanism(s) of gymnastics injury and the influence of different landing techniques on injury prevention should be considered in counter measure research. More recently, the continued need to 'bridge the gap' between laboratory identification of biomechanical risk factors in landing and clinical practice has been recognised by Myer and colleagues [39].

### Theoretical studies of loading mechanisms

Despite intense laboratory-based study, the associated descriptive analyses, and control and ethical constraints has precluded a thorough causal insight into the effects of prevalent loading mechanisms in challenging impact landings. As indicated in Table 1, biomechanics studies using a less conventional theoretical approach have emerged in the landing literature. A theoretical approach uses a theory or model to make predictions about the behaviour of a system [40] and permits a non-invasive, systematic manipulation of independent variables or mechanisms of interest through simulation. While a theoretical approach alone offers a solution to the limited control associated with more traditional scientific approaches, caution in using the approach must be taken due to a potential lack of realism to the living human performer. Laboratory-derived data from experimental or observational studies are however frequently used to ensure realistic inputs for a theoretical model, and to check the accuracy and validity of the predicted outcomes.

Until recently, theoretical approaches using rigid body simulation models, which assume the human performer may be represented by a series of single segment rigid components, have been customary for gaining insight into loading mechanisms [41,42]. The frequent presence of uncharacteristic oscillations in the internal joint load estimations derived in numerous kinetic analyses conducted using rigid body and inverse dynamic assumptions [16,22] had however questioned the assumption of whole body rigidity, particularly for dynamic impacts. In 1998, Gruber, and colleagues [43] conducted an innovative theoretical study to specifically examine the potential limitations of inverse dynamic analyses and rigid body assumptions on load estimations during a gymnastic-style drop landing. Gruber and colleagues [43] reported that rigid body assumptions yielded completely incorrect predictions of internal joint loads during the impact phase such that hip joint torques may be three to four times too large. More realistic skeleto-mechanical models of impact landings that incorporate soft tissue properties have subsequently become more evident in the literature over the past decade. The models, which have been termed 'wobbling mass' models, have increasingly been used to investigate the influence of soft and rigid tissue mass compositions on impact loading during simulated running [44,45] and more dynamic gymnastic-style landings [20,46-49]. Simulation studies employing 'wobbling mass' models have reported soft tissue contributions to an external peak impact load reduction of as much as 8.6 [49] and 24.3 [19] bodyweights in double foot drop landings performed with a forefoot- and heel-first ground contact, respectively.

'Wobbling mass' studies have partially attributed soft tissue loading contributions to regulation of the coupling between soft and rigid masses, which has been associated with modified muscle activity levels or muscle tuning [45]. Mass coupling represents the elasticity and damping characteristics of soft tissue (e.g. muscle, skin, subcutaneous fat) motion relative to the underlying rigid mass (e.g. bone) within a human segment e.g. thigh or shank. Liu and Nigg [44] had further supported the notion that through muscle tuning, mass coupling properties may interact with inherent mass distributions to control the forces incurred in simulated running impacts. Reductions in the damping between soft and rigid masses were later found to positively interact with localised rigid mass compositions by providing a 0.13 bodyweight additional external load attenuation during potentially injurious gymnastic-style landings [20]. If the concept of muscle tuning is correct, a subject-specific response to impact loading may be expected [45] and has been supported by the reporting of idiosyncratic responses to peak load attenuation with adapted soft tissue compositions [20]. Insight into prevalent loading mechanisms and injury predisposition in gymnastic-style landings may subsequently warrant more individual performer analyses in the future.

The theoretical support for a link between impact loading and mass compositions advocates the need for extended 'wobbling mass' modelling research into regulatory mechanisms that may alleviate a performer's innate injury predisposition. While high-speed filming of impact situations has provided insight into the complex damped manner of soft tissue motion [43], the general lack of soft and rigid mass information from living subjects currently inhibits the widespread use of 'wobbling mass' models [50]. While evidently beneficial, the increase in model complexity associated with wobbling mass compared to rigid body models is further associated with heightened development and processing demands. Despite the potential increase in simulation run time, Mills and colleagues [47] advocated a continued need to prioritise the selection of a simulation model that determines realistic internal forces when assessing injury risk in gymnastics landing. The continued merger of laboratory information with advances in theoretical modelling may subsequently offer a successful approach to ensuring a sustained enhancement of knowledge in impact biomechanics and injury prevention strategies.

### Application of theoretical studies to injury prevention strategies

While recent efforts to integrate laboratory-based findings into injury prevention studies are being made [37-39], the explicit integration of theoretical study insights remains sparse. Unlike laboratory-based studies, theoretical investigations can provide explicit information on the magnitude of impact loading change that may be incurred by systematic changes to a regulatory loading mechanism. The permitted address of 'cause and effect' based research questions can subsequently provide advance knowledge for injury prevention interventions.

Recent theoretical studies [19,49] have explicitly supported the load attenuation benefits of soft tissue properties and marginally adapted mass coupling regulatory mechanisms, which have traditionally been difficult to ascertain in laboratory-based studies. Further studies have acknowledged that while a performer's innate mass compositions may be difficult to alter, tuning of the self-selected landing technique [51] and mass coupling [19,49] responses may alleviate a natural predisposition to high impact loading. As further suggested in the study of Gittoes and colleagues [51], marginal changes (up to 5 ms) to the timing of the ankle and knee joint action could influence external impact loading by as much as 3.9 and 1.5 bodyweights, respectively in gymnastic-style landing protocols.

Due the potential counter effect of internal and external loading [48,51], and idiosyncratic responses [51], caution in considering the explicit influence of regulatory changes on impact loading has been highlighted by the theoretical literature. As recently evidenced by Mills and colleagues [48], using a reduction in external loading (ground reaction force), due to a change in landing technique, as a basis for a reduction in injury potential in gymnastic movements may not be appropriate since internal loading can be heightened. Injury prevention programmes that are customised to specific performers, and assessed following internal and external loading analyses may therefore be warranted when informing prevention strategies developed to alleviate the physical demands incurred in gymnastic-style landings.

### Future directions

While the existing body of biomechanical research into gymnastic-style impact landings has traditionally been laboratory-based, theoretical studies, which have offered distinct advancements in knowledge, remain relatively sparse in the literature. In order to benefit from the ecological validity of laboratory studies, and the systematic control and non-invasive testing environment of theoretical investigations, a more widespread use of an approach that integrates data obtained from the field or laboratory with theoretical models may be advocated in future investigations. In particular, the growing number of theoretical studies using 'wobbling mass' models [20,46,47], may be more effectively employed to develop insight into loading in gymnastic-style landings with the availability of increased empirical support regarding soft tissue properties. While the mechanisms predisposing a performer to gymnastic-style impact injuries may be multi-factorial in nature, the majority of existing research continues to favour examination of isolated loading mechanisms. The extent to which regulatory mechanisms can be used to alleviate an innate predisposition remains relatively under-documented. Evidence of performer-specific responses to impact loading [20,31] supports a need to further consider, through the simultaneous examination of multiple mechanisms, the interaction of innate profiles and regulatory mechanisms in understanding injury predisposition. A deviation from the tendency to consider internal and external loading in isolation may be further advocated in future research due to evidential support from theoretical studies [48,51] for a potential antagonistic response in the respective measures.

Considering the likely maintenance of a scoring system for gymnastic dismounting, which requires the achievement of constrained landing techniques, regulatory strategies such as mass coupling tuning, may alleviate a performer's innate predisposition to high physical demands without substantial alterations to technique. Accommodating self-selected landing techniques, which are tailored to the movement conditions and a performer's unique physical composition in the scoring system, may conversely offer substantially greater protection benefits for performers repeatedly executing demanding landings. The success of injury referral schemes and clinical practice is partially reliant on the comprehensive evaluation of injury prevention programmes used in training and competition. While the suggested need for a growing body of scientific evidence remains justified [6], further attempts to fully integrate existing insight in the development and evaluation of tailored injury prevention interventions is also warranted.

## Conclusion

The review has appraised the development of current understanding of the loading mechanisms contributing to the physical demands of gymnastic-style impact landings using biomechanical approaches. While current insights have typically been derived from laboratory-based studies, investigations employing theoretical approaches are becoming more widely employed in the literature. As a partial consequence of the tendency to examine isolated mechanisms within the respective laboratory and theoretical studies, the primary loading mechanisms influencing the physical demands of gymnastic-style landings remain difficult to ascertain. While enhanced scientific understanding of the interaction of inherent and regulatory mechanisms is warranted to inform potential scoring changes in gymnastics, increased attempts to inform the development and evaluation of tailored injury prevention interventions using existing insights should also be made.

## Competing interests

The authors declare that they have no competing interests.

## Authors' contributions

MJRG prepared and compiled the manuscript. MJRG and GI both contributed to the appraisal of the literature and the approval of the final manuscript.
